# Socio-demographic and community-level determinants of delayed breastfeeding initiation in Borama, Somalia: a multilevel mixed-effects analysis

**DOI:** 10.3389/fnut.2026.1945737

**Published:** 2026-09-30

**Authors:** Yusuf Abdi Hared, Abdirahman Omer Ali, Abdisalam Mahdi Hassan, Hodan Saed Ahmed, Muhiadin Ismail Abdi, Hodo Abdi Abdulahi, Saralees Nadarajah

**Affiliations:** 1 Center for Community Services, Amoud University, Borama, Somalia; 2 School of Health and Welfare, Dalarna University, Falun, Sweden; 3 College of Health Sciences, Amoud University, Borama, Somalia; 4 Department of Mathematics, University of Manchester, Manchester, United Kingdom

**Keywords:** exclusive breastfeeding, infant nutrition, multilevel analysis, risk factors, Somalia

## Abstract

**Background:**

Timely initiation of breastfeeding is a proven life-saving intervention for reducing neonatal morbidity and mortality. Despite global advocacy, adherence in fragile health systems remains critically low. This study assesses the magnitude and multi-level epidemiological predictors of delayed breastfeeding initiation among postnatal mothers in Borama, Somalia.

**Methods:**

An institution-based cross-sectional study was conducted among 392 postnatal mothers (aged 15–49 years) recruited via systematic random sampling at the Sheekh Osman Center for Community Health. Data were gathered using a pre-tested, structured interviewer-administered questionnaire. Delayed breastfeeding initiation was defined as initiating suckling more than 30 min postpartum. Participants were nested within 14 recognized municipal neighborhood clusters in Borama. To account for community-level clustering, a four-step hierarchical multilevel mixed-effects logistic regression was performed. Fixed effects were expressed as Adjusted Odds Ratios (aOR) with 95% Confidence Intervals (CI), and random effects were quantified using the Intraclass Correlation Coefficient (ICC).

**Results:**

The magnitude of delayed breastfeeding initiation was 40.8% (95% CI: 35.9–45.8%; n=160 ). In bivariate analysis, delayed initiation was significantly associated with maternal education level ( p<0.001 ) and absence of antenatal breastfeeding information ( p<0.001 ). In the final fully adjusted multilevel model, the absence of breastfeeding counseling during antenatal care was the strongest independent predictor of delay (aOR = 7.93, 95% CI: 4.51–13.95, p<0.001 ). Mothers without formal education had three-fold higher odds of delayed initiation compared to college-educated mothers (aOR = 3.07, 95% CI: 1.33–7.07, p=0.008 ). Mothers aged 15–20 years had lower odds of delay compared to those older than 30 years (aOR = 0.39, 95% CI: 0.18–0.87, p=0.021 ). The null model demonstrated significant community clustering (ICC = 18.1%, p<0.001 ), which increased to 25.6% in the final adjusted model.

**Conclusion:**

Over four in ten mothers experienced delayed breastfeeding initiation. Gaps in antenatal counseling, lack of maternal formal education, and neighborhood-level clustering are primary drivers of delayed initiation. Health authorities must implement facility-based Baby-Friendly Hospital Initiative protocols and intensify antenatal lactation counseling.

## 1 Introduction

Breastfeeding constitutes the biological cornerstone of neonatal nutrition, serving as a critical determinant of survival, growth, and development during the early postnatal period (1). Early initiation of breastfeeding (EIBF) and exclusive breastfeeding (EBF) for the first 6 months are recognized globally as essential preventive interventions against neonatal and infant morbidity and mortality (2, 3). These optimal feeding practices align directly with United Nations Sustainable Development Goals (SDG 2 and SDG 3) and the African Union Agenda 2063 (4, 5). Biochemically, colostrum supplies essential immunoglobulins, bioactive enzymes, and oligosaccharides that reduce the incidence of gastrointestinal infections, neonatal sepsis, and respiratory tract illnesses (3, 6).

Globally, only approximately 40% of infants under 6 months are exclusively breastfed (1, 7). In Sub-Saharan Africa, while breastfeeding is near-universal, rates of early initiation vary substantially due to systemic, clinical, and cultural barriers (8, 9). In East Africa, disparities between urban and rural settings remain prevalent (3, 10). In Somalia and Somaliland, where maternal and child health infrastructure has been strained by decades of socio-political vulnerability, infant feeding indicators remain critically low; national demographic surveys report that only 26% of neonates are put to the breast within the first hour of life, accompanied by widespread colostrum discarding and early administration of pre-lacteal feeds such as sugar solutions, tea, and animal milk (11).

While global guidelines established by the World Health Organization (WHO) and UNICEF benchmark early initiation within the first 60 min of delivery (1), contemporary clinical quality improvement initiatives and immediate skin-to-skin care protocols advocate for uninterrupted suckling initiation within the first 30 min postpartum to maximize maternal oxytocin release, reduce postpartum hemorrhage, and facilitate immediate neonatal metabolic transition (2, 6, 12).

Infant feeding behaviors are shaped by complex individual, behavioral, and structural factors (12–14). However, localized epidemiological studies from transitional urban centers in Somalia remain scarce (11). Borama, an academic and economic center in the Awdal region of Somaliland/Somalia, exhibits unique socio-demographic patterns with rapid peri-urban growth. Evaluating individual and neighborhood-level determinants using multilevel modeling is essential for identifying actionable targets for public health intervention. This study assesses the magnitude and multi-level epidemiological predictors of delayed breastfeeding initiation among postnatal mothers seeking care at the Sheekh Osman Center for Community Health in Borama, Somalia.

## 2 Methodology

### 2.1 Study design and setting

An institution-based cross-sectional epidemiological study was conducted at the Sheekh Osman Center for Community Health in Borama, Somalia. The facility is managed by the College of Medicine and Health Sciences at Amoud University and provides specialized primary healthcare, maternal and child health (MCH) services, and routine obstetric care to urban and peri-urban catchment communities. Ethical clearance was approved by the Research Ethics Committee (Reference number: SLMA/REC/2026/003). Written informed consent was obtained from all participants prior to enrollment.

### 2.2 Study population and sampling strategy

The study population included postnatal mothers aged 15–49 years who had delivered a live birth via uncomplicated vaginal delivery at the center within the preceding 72 h.

Mothers with multiple gestations (e.g., twins or triplets) were excluded from this study because multiple births involve distinct clinical complications, elevated rates of neonatal intensive care admission, low birth weight, and prolonged physical exhaustion, all of which independently disrupt routine postpartum feeding timelines. Additionally, mothers whose neonates required immediate neonatal intensive care unit (NICU) admission, those with severe postpartum maternal illness, and transient visitors residing outside Borama were excluded.

The sample size was computed using Cochran’s single population proportion formula:

$$n=\frac{Z^{2}\cdot p(1-p)}{d^{2}}$$

Where Z=1.96 (95% confidence level) and margin of error d=0.05 . A conservative expected proportion of p=0.50 was used to ensure maximum sample size and statistical power, given the wide variation in reported early feeding indicators across Somali regions. Because the annual delivery catchment population at the facility exceeds 10,000 deliveries ( n/N<0.05 ), finite population correction was not required. Accounting for potential non-response, 392 eligible mothers were recruited using systematic random sampling from the facility’s daily postnatal admission log.

Participants were grouped into 14 distinct residential neighborhood clusters (*xaafado*) within Borama municipality (Sheikh Osman, Sheikh Ali Jowhar, Sheikh Makahiil, Halane, Rayd, Qandhada, Goljano, 1 July, Sh. Nuur, Sh. Cumar, Stadium, Xalane-B, Barwaaqo, and Camuud peri-urban sector). The number of participants per neighborhood cluster ranged from 18 to 41, with an average cluster size of 28.0 mothers ( SD=6.4 ), satisfying requirements for stable estimation of two-level random intercept parameters.

For the analytical multilevel logistic regression, the sample size of 392 mothers with 160 outcome events provided over 22 events per variable (EPV) across all model parameters, exceeding standard sample size criteria (EPV ≥10 ) and ensuring >80% power to detect adjusted odds ratios ≥1.8 at α=0.05 .

### 2.3 Variables and operational definitions

- Primary Outcome Variable: Delayed Initiation of Breastfeeding, operationalized as the initiation of suckling more than 30 min postpartum (Binary: 1=Delayed[>30minutes] , 0=Timely[≤30minutes] ). This operational threshold aligns with facility-level clinical monitoring standards for immediate skin-to-skin contact (12).
- Level-1 Individual Covariates: Maternal age (15–20, 21–25, 26–30, >30 years), maternal educational attainment (none, primary, secondary, higher/college), employment status (housewife vs. employed/business), marital status (married vs. single/divorced/separated), and antenatal care (ANC) breastfeeding counseling (measured as receiving structured verbal or visual information on early suckling, colostrum, and positioning from healthcare providers during antenatal visits: Yes vs. No).
- Level-2 Clustering Variable: Participant residential community/neighborhood cluster within Borama municipality (14 discrete administrative neighborhood units defined by municipal borders).

### 2.4 Data collection and quality assurance

Data were gathered through face-to-face interviews using a pre-tested, structured questionnaire. Five qualified female midwives and community health researchers fluent in Somali conducted the interviews. The instrument was pre-tested on a 5% pilot sample ( n=20 ) in an adjacent maternal health clinic to ensure conceptual clarity and cultural validity; pilot data were excluded from the final analysis. Data collectors underwent 2 days of standardized training on interview techniques, ethical conduct, and objective recording of delivery and feeding times. Completed questionnaires were inspected daily by the field supervisor for missing values, discrepancies, and entry errors. Cleaned data were double-entered into EpiData version 4.6 and exported to Stata version 17.0 and R version 4.3 for statistical modeling.

### 2.5 Step-by-step statistical modeling process

Data analysis followed a predefined, reproducible four-step protocol:

1. Descriptive Summary: Categorical variables were summarized as absolute frequencies and percentages. The 95% confidence interval for the primary outcome prevalence was calculated using the Wilson score method.
2. Bivariate Analysis: Pearson’s Chi-square ( $\chi^{2}$ ) tests were performed to evaluate crude associations between individual characteristics and delayed breastfeeding initiation. Statistical significance was set at two-sided p<0.05 .
3. Multicollinearity Diagnostics: Variance Inflation Factors (VIF) and tolerance statistics were evaluated across all candidate covariates; all VIF values were <1.8 , confirming no collinearity. All individual VIF values were <1.45 (maternal education VIF=1.38 ; occupational status VIF=1.25 ; maternal age VIF=1.41 ; marital status VIF=1.08 ; ANC counseling VIF=1.15 ), with a mean VIF of 1.25, confirming the absence of multicollinearity.
4. Multilevel Logistic Regression Modeling: A two-level hierarchical mixed-effects model was specified with mothers ( i ) nested within community clusters ( j ):
$$\text{logit}(p_{\mathrm{ij}})=\beta_{0}+\beta^{T}X_{\mathrm{ij}}+u_{j},\;\;u_{j}∼N(0,\sigma_{u}^{2})$$

Four sequential nested models were estimated: Model 0 (Null/Empty Model): Included only the random cluster intercept ( $u_{j}$ ) to evaluate community-level clustering and baseline Intraclass Correlation Coefficient (ICC). Model 1: Adjusted for individual-level maternal socio-demographic covariates (age, education, marital status, employment). Model 2: Adjusted for the structural health service variable (antenatal breastfeeding counseling). Model 3 (Final Full Model): Combined all individual socio-demographic and structural healthcare predictors.

Sensitivity Analysis: To evaluate the potential confounding influence of parity on the estimated associations, a sensitivity analysis was conducted on a sub-cohort of mothers with documented gravidity/parity records ( n=312 ). Including parity (primiparous vs. multiparous) did not meaningfully alter the magnitude or statistical significance of the primary predictors: the adjusted odds ratio for lack of antenatal counseling remained aOR=7.41 (95% CI: 3.98–13.79, p<0.001 ), and no formal education remained aOR=2.94 (95% CI: 1.24–6.96, p=0.014 ), confirming the robustness of the primary model.

Goodness-of-fit and model comparisons were evaluated using −2 Log-Likelihood (Deviance), Akaike Information Criterion (AIC), Bayesian Information Criterion (BIC), and Likelihood Ratio (LR) tests versus standard single-level logistic regression.

## 3 Results

### 3.1 Cohort characteristics and prevalence of delayed initiation

A total of 392 postnatal mothers were included in the final analysis. The cohort was distributed across maternal age categories as follows: 115 mothers (29.3%) were aged 15–20 years, 107 (27.3%) were aged 21–25 years, 100 (25.5%) were aged 26–30 years, and 70 (17.9%) were aged >30 years. Regarding marital status, 331 mothers (84.4%) were married, and 61 (15.6%) were single, divorced, or separated. In terms of occupation, 303 mothers (77.3%) were housewives, and 89 (22.7%) were employed or running a business. Regarding educational attainment, 143 mothers (36.5%) had no formal education, 118 (30.1%) completed primary education, 80 (20.4%) completed secondary education, and 51 (13.0%) completed higher/college education. During antenatal care visits, 252 mothers (64.3%) received breastfeeding information, while 140 (35.7%) did not receive any counseling.

The overall prevalence of delayed breastfeeding initiation ( >30 minutes postpartum) was 40.8% (95% CI: 35.9–45.8%; n=160 ), while 59.2% (95% CI: 54.2–64.1%; n=232 ) initiated breastfeeding within 30 min of birth.

### 3.2 Bivariate analysis of factors associated with breastfeeding initiation

Table 1 displays the socio-demographic and healthcare-related characteristics of the 392 mothers cross-tabulated by timing of breastfeeding initiation.

**Table 1:** Socio-demographic and clinical characteristics associated with breastfeeding initiation timing ( N=392 ).

Characteristic	Total cohort, n (%)	Timely initiation ( ≤30 min), n (%) [ n=232 ]	Delayed initiation ( >30 min), n (%) [ n=160 ]	$\chi^{2}$ value	p -value^†^
Maternal age (years)				1.68	0.64
>30	70 (17.9%)	41 (17.7%)	29 (18.1%)		
15–20	115 (29.3%)	68 (29.3%)	47 (29.4%)		
21–25	107 (27.3%)	64 (27.6%)	43 (26.9%)		
26–30	100 (25.5%)	59 (25.4%)	41 (25.6%)		
Highest education				30.12	<0.001
Higher/College	51 (13.0%)	41 (17.7%)	10 (6.3%)		
Secondary	80 (20.4%)	54 (23.3%)	26 (16.3%)		
Primary	118 (30.1%)	75 (32.3%)	43 (26.9%)		
No formal education	143 (36.5%)	62 (26.7%)	81 (50.6%)		
Occupational status				1.12	0.29
Employed/Business	89 (22.7%)	56 (24.1%)	33 (20.6%)		
Housewife	303 (77.3%)	176 (75.9%)	127 (79.4%)		
Marital status				0.06	0.80
Married	331 (84.4%)	196 (84.5%)	135 (84.4%)		
Single/Divorced/Sep.	61 (15.6%)	36 (15.5%)	25 (15.6%)		
ANC breastfeeding Info				47.38	<0.001
Received (Yes)	252 (64.3%)	181 (78.0%)	71 (44.4%)		
Not received (No)	140 (35.7%)	51 (22.0%)	89 (55.6%)		

^†^ Pearson’s Chi-squared test.

Bivariate analysis identified statistically significant associations between delayed breastfeeding initiation and both maternal education level ( $\chi^{2}=30.12,p<0.001$ ) and absence of breastfeeding information during ANC ( $\chi^{2}=47.38,p<0.001$ ). Among mothers who delayed initiation, 50.6% ( n=81 ) had no formal education, compared to 26.7% ( n=62 ) among those with timely initiation. Similarly, 55.6% ( n=89 ) of mothers with delayed initiation had received no antenatal breastfeeding counseling, compared to 22.0% ( n=51 ) in the timely initiation group. Maternal age ( p=0.64 ), occupation ( p=0.29 ), and marital status ( p=0.80 ) were not statistically significant in unadjusted bivariate testing.

### 3.3 Multilevel mixed-effects logistic regression analysis

Table 2 presents the results of the hierarchical multilevel mixed-effects models.

**Table 2:** Multilevel mixed-effects logistic regression models and fit diagnostics ( N=392 ).

Variables	Category	Model 0 (Null model)	Model I (aOR, 95% CI)	Model II (aOR, 95% CI)	Model III (Final model) (aOR, 95% CI)
Maternal age	>30 Years	–	Reference	–	Reference
	15–20 Years	–	0.47 (0.23–0.97)*	–	0.39 (0.18–0.87)*
	21–25 Years	–	0.69 (0.34–1.39)	–	0.60 (0.28–1.30)
	26–30 Years	–	0.73 (0.36–1.48)	–	0.70 (0.32–1.56)
Marital status	Married	–	Reference	–	Reference
	Single/Divorced	–	1.19 (0.64–2.20)	–	0.99 (0.50–1.95)
Education level	Higher/College	–	Reference	–	Reference
	Secondary	–	1.08 (0.45–2.58)	–	0.88 (0.33–2.29)
	Primary	–	0.69 (0.31–1.55)	–	0.75 (0.31–1.82)
	No education	–	2.41 (1.13–5.14)*	–	3.07 (1.33–7.07)*
Occupation	Employed/Business	–	Reference	–	Reference
	Housewife	–	1.85 (1.04–3.29)*	–	1.60 (0.86–2.96)
ANC counseling	Received info (Yes)	–	–	Reference	Reference
	No info (No)	–	–	6.46 (3.89–10.71)*	7.93 (4.51–13.95)*
Random effects & model fit statistics					
$\tau^{2}$ (Cluster variance)		0.725 ( p<0.001 )	0.790 ( p<0.001 )	0.977 ( p<0.001 )	1.130 ( p<0.001 )
ICC (%)		18.1%	19.4%	22.9%	25.6%
LR test vs. Logistic regression		–	$\chi^{2}=34.45,p<0.001$	–	$\chi^{2}=36.50,p<0.001$
Deviance ( −2LL )		482.61	448.16	423.37	**386.88**
AIC		486.61	468.16	429.37	**408.88**
BIC		494.55	507.87	441.29	452.56

^*^ Statistically significant at p<0.05 . aOR, Adjusted Odds Ratio; CI, Confidence Interval; ICC, Intraclass Correlation Coefficient; AIC, Akaike Information Criterion; BIC, Bayesian Information Criterion. Bold values indicate statistically significant associations (*p* < 0.05). AOR, Adjusted Odds Ratio; CI, Confidence Interval; ICC, Intraclass Correlation Coefficient; AIC, Akaike Information Criterion; BIC, Bayesian Information Criterion.

In Model I (adjusting for individual socio-demographics), maternal age 15–20 years was associated with lower odds of delay compared to age >30 years (aOR = 0.47, 95% CI: 0.23–0.97, p=0.042 ). Mothers with no formal education exhibited higher odds of delay compared to college-educated mothers (aOR = 2.41, 95% CI: 1.13–5.14, p=0.023 ). Housewives showed elevated odds of delay in Model I (aOR = 1.85, 95% CI: 1.04–3.29, p=0.036 ).

In Model II (adjusting for health service exposure alone), lack of ANC breastfeeding information was associated with more than six-fold higher odds of delay (aOR = 6.46, 95% CI: 3.89–10.71, p<0.001 ).

In the final fully adjusted mixed-effects model (Model III), three variables remained independently associated with delayed initiation: 1. Antenatal Counseling: Mothers who received no breastfeeding information during ANC visits had nearly eight times higher odds of delayed initiation compared to those who received counseling (aOR = 7.93, 95% CI: 4.51–13.95, p<0.001 ). 2. Maternal Education: Mothers with no formal education were three times more likely to delay initiation compared to college-educated mothers (aOR = 3.07, 95% CI: 1.33–7.07, p=0.008 ). 3. Maternal Age: Mothers aged 15–20 years had 61% lower odds of delayed initiation compared to mothers aged >30 years (aOR = 0.39, 95% CI: 0.18–0.87, p=0.021 ).

Occupational status (housewife: aOR = 1.60, 95% CI: 0.86–2.96, p=0.138 ) and marital status (single/divorced: aOR = 0.99, 95% CI: 0.50–1.95, p=0.978 ) showed no statistically significant association in the final model.

### 3.4 Community random effects and model evaluation

The null model demonstrated significant community-level random variance ( $\tau^{2}=0.725,p<0.001$ ), corresponding to an ICC of 18.1%. This confirms that nearly one-fifth of the baseline variation in delayed initiation was attributable to neighborhood clustering.

When adjusting for individual socio-demographics and ANC counseling, the unexplained cluster variance increased to $\tau^{2}=1.130$ (ICC = 25.6%). The Likelihood Ratio test comparing the full multilevel model against standard single-level logistic regression was highly significant ( $\chi^{2}=36.50,p<0.001$ ). Model III demonstrated the lowest deviance (386.88) and lowest AIC (408.88), indicating superior model fit.

## 4 Discussion

In this cross-sectional study in Borama, Somalia, 40.8% of postnatal mothers delayed initiating breastfeeding beyond the target of 30 min postpartum. The findings demonstrate that delayed initiation is jointly driven by individual socio-demographic factors, structural factors in health service delivery, and the residential community context.

The single strongest predictor of delayed initiation was the lack of breastfeeding counseling during antenatal care visits ( aOR=7.93 , 95% CI: 4.51–13.95). The substantial magnitude of this adjusted odds ratio highlights that structured antenatal lactation education serves as a pivotal clinical gateway for early feeding practices. Antenatal counseling equips mothers with physiological knowledge regarding colostrum let-down, builds maternal self-efficacy, and directly dispels persistent traditional beliefs—such as the perception that colostrum is “dirty” or toxic and must be discarded, or that newborns require pre-lacteal warm water or clarified butter (subag) before mature milk arrives (11, 15, 19, 21, 22). When this structural educational touchpoint is omitted during pregnancy, mothers are left without clinical guidance and are far more prone to defer feeding until hours after delivery.

Nonetheless, the magnitude of this association ( aOR≈8 ) should be interpreted with appropriate epidemiological caution. While it indicates that antenatal counseling is a high-leverage clinical intervention, this parameter likely reflects unmeasured confounding: mothers who actively seek or recall receiving antenatal breastfeeding counseling may possess higher intrinsic health-seeking motivation, attend a greater total number of comprehensive ANC visits, have higher overall health literacy, and receive better social support. In cross-sectional designs, the odds ratio overstates the relative risk when the outcome is common ( 40.8% ). Therefore, while counseling may not independently reduce delay by an absolute factor of eight, it remains an indispensable, actionable intervention within the maternal healthcare delivery package (9, 16, 20).

Maternal education was another strong independent determinant, with illiterate mothers exhibiting three times the odds of delayed initiation compared to college-educated peers (aOR = 3.07). Maternal formal education enhances health literacy, facilitates comprehension of clinical guidance, and empowers mothers to adhere to early initiation protocols despite contradictory domestic advice (2, 8, 12).

Our findings regarding maternal age indicate that younger mothers aged 15–20 years had 61% lower odds of delayed initiation (aOR = 0.39) compared to mothers aged over 30. While some regional studies report that adolescent mothers experience feeding challenges due to inexperience (8, 17), younger mothers in urban Somali settings may be more receptive to clinical guidance and health promotion messaging delivered by midwives during facility visits (11). In contrast, older multiparous mothers may adhere to established domestic feeding practices, rely on elder matriarchal guidance, or experience greater physical fatigue during labor, contributing to delayed suckling (11, 18).

Multilevel diagnostics revealed an ICC of 25.6% in the fully adjusted model. This high degree of community-level clustering indicates that infant feeding practices in Borama are strongly influenced by shared neighborhood environments, localized peer norms, and variations in community midwife access (10, 11).

### 4.1 Strengths and limitations

#### 4.1.1 Strengths

This study is among the first in Somalia to utilize a two-level mixed-effects logistic regression framework to account for neighborhood clustering in infant feeding practices. The systematic sampling strategy minimized selection bias within the institutional cohort.

#### 4.1.2 Limitations

First, the cross-sectional design precludes establishing direct temporal causality. Second, feeding timing was determined via maternal recall within 72 h postpartum, which may introduce minor recall inaccuracies. Third, the measurement of antenatal counseling was binary (Yes/No) and did not evaluate counseling duration, curriculum content, provider credentials, or message retention. Fourth, the analysis was subject to unmeasured clinical confounding: detailed labor records—including exact parity, labor duration, use of oxytocin augmentation, and perineal trauma (episiotomy or severe tears)—as well as neonatal birth weight, were not routinely captured in the outpatient delivery register. While our sensitivity analysis in a sub-sample with parity data confirmed the robustness of our primary findings, unmeasured clinical and socioeconomic variables could explain additional variance. Finally, because data collection occurred at a single urban teaching health center, findings may not fully generalize to remote rural pastoralist communities.

### 4.2 Policy and clinical implications

1. Facility-Based Implementation: Maternity centers should systematically institutionalize the Baby-Friendly Hospital Initiative (BFHI), enforcing immediate, uninterrupted skin-to-skin contact for all stable vaginal deliveries.
2. Antenatal Standardization: Antenatal care packages must integrate mandatory, culturally tailored lactation education sessions delivered in low-literacy Somali formats.
3. Community-Level Outreach: Public health agencies must deploy localized community health worker programs to address neighborhood-specific cultural beliefs regarding colostrum and pre-lacteal feeds.

## 5 Conclusion

Delayed initiation of breastfeeding affects 40.8% of vaginally delivered newborns at the Sheekh Osman Center for Community Health in Borama, Somalia. Absence of antenatal breastfeeding counseling and lack of maternal formal education are the primary independent risk factors, with significant clustering across residential municipal neighborhoods. Younger maternal age demonstrated a protective association against delayed initiation. Interventions must focus on eliminating missed educational opportunities during routine antenatal visits, expanding low-literacy maternal lactation counseling, and enforcing immediate postpartum skin-to-skin contact protocols within maternity centers.

## Data Availability

The original contributions presented in the study are included in the article/supplementary material, further inquiries can be directed to the corresponding author.
